# Focused Ultrasound Therapies for Brain, Liver, Prostate, and Breast Cancer Applications: Clinical Evidence and Future Directions

**DOI:** 10.3390/cancers18172890

**Published:** 2026-09-07

**Authors:** Nassib Abou Heidar, Daniel Sheeran, Divine Nwafor, Olivia C. Sears, Shayan Moosa, Lynn T. Dengel, Kirsten Greene, Alan H. Matsumoto

**Affiliations:** 1 Department of Urology, University of Virginia Health, Charlottesville, VA 22908, USA; 2 Department of Radiology and Medical Imaging, University of Virginia Health, Charlottesville, VA 22908, USA; dps7u@uvahealth.org; 3 Department of Neurosurgery, University of Virginia Health, Charlottesville, VA 22908, USA; 4 Department of Surgery, University of Virginia Health, Charlottesville, VA 22908, USA

**Keywords:** focused ultrasound, HIFU, clinical trials, histotripsy, locoregional therapy, drug delivery, immunotherapy, sonodynamic therapy, blood–brain barrier opening, cancer treatment, noninvasive image-guided therapy

## Abstract

Focused ultrasound (FUS) is an emerging and evolving minimally invasive therapeutic platform that is helping to redefine the management of malignant diseases. Clinical translation is advancing rapidly, with several completed and ongoing clinical trials demonstrating the safety, feasibility, and early efficacy of FUS in the treatment of cancer. This manuscript provides a synthesis of some of the available clinical data and device platforms being used and evaluates the results from completed and active clinical trials for cancers involving four different solid organs. To stay within the editorial parameters of the invited article, this review will not provide a meta-analysis of clinical trials data and/or the literature but rather highlight some of the currently available clinical evidence and FUS technologies that have been used in the treatment of cancers of the brain, liver, prostate gland, and breast.

## Simple Summary

Focused ultrasound (FUS) is a novel, noninvasive, image-guided technology that uses focused and precisely targeted sound waves to treat cancer without incisions or use of ionizing radiation. Depending on how the ultrasound energy is delivered, FUS can potentially destroy tumors through heat or mechanical forces, improve the delivery of drugs and immunotherapies to tumors, and enhance anti-cancer immune responses. Clinical trials have also demonstrated the safety and feasibility of applying FUS in the treatment of a variety of cancers. This brief review article provides a glimpse into and will highlight some of the current clinical evidence, available technologies (both FDA and non-FDA-approved), and emerging applications of FUS for the treatment of brain, liver, prostate and breast cancers.

## 1. Introduction

The management of cancers remains constrained by a complex interplay of biological, anatomical, immunological, and therapeutic challenges, limiting the efficacy of conventional therapies despite substantial advances in surgery, systemic and image-guided therapies, and radiation oncology [1]. The emergence of focused ultrasound (FUS) as a noninvasive therapy has generated considerable interest due to its potential to overcome many of these challenges. FUS offers a fundamentally different approach to other traditional cancer therapies by the use of image-guided and noninvasive delivery of acoustic energy to precise locations to generate specific and varying biological effects without the need for incisions or use of ionizing radiation. Importantly, the effects of FUS are not limited to thermal ablation of tissue alone. Depending on the technical parameters used, FUS can increase vascular permeability, facilitate localized drug activity/delivery with sonodynamic therapy (SDT) or sonoporation of tight cell junctions, create cavitation-mediated cellular liquefaction, and/or stimulate immune responses. This manuscript reviews the technological foundations, biological mechanisms, clinical trial evidence, and future directions of FUS therapies for malignancies of the brain, liver, prostate gland, and breast.

## 2. Brain Cancers

### 2.1. Methods and References Rationale

We searched ClinicalTrials.gov, the largest public registry of interventional oncology trials, in June 2026 using the search terms “glioblastoma AND focused ultrasound,” “glioma AND focused ultrasound,” “brain AND focused ultrasound,” “blood–brain barrier AND focused ultrasound,” and “brain metastases AND focused ultrasound,” restricted to interventional trials in which a focused ultrasound (FUS)- or high-intensity focused ultrasound (HIFU)-based device was used as a study intervention. Trials were included regardless of recruitment status (completed, recruiting, or active-not-recruiting) if they enrolled patients with a primary or metastatic brain tumor, or if FUS was used as a diagnostic (liquid-biopsy) modality in this population. Duplicate registrations and trials in which ultrasound served only as an imaging-guidance tool for another therapy were excluded. We note the limitations of this approach: it was not a formal systematic review with dual independent screening, it is restricted to single registries and does not capture trials registered exclusively in other national registries (e.g., the EU Clinical Trials Register), and it reflects a single time point (June 2026); so, the trial status may have since changed from that time point.

### 2.2. Devices Currently in Clinical Use

**Figure 1:**
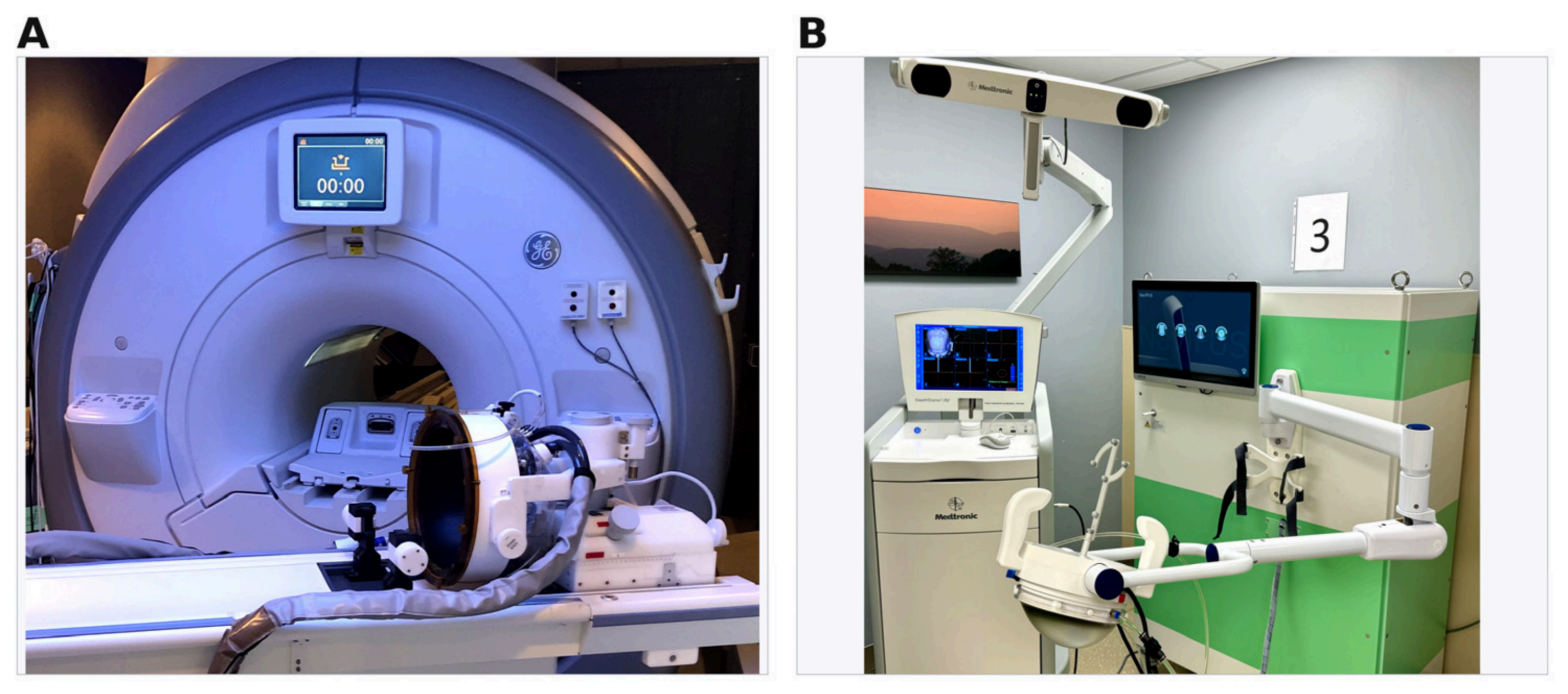
Focused ultrasound technology. Representative focused ultrasound (FUS) technologies used in active brain-cancer clinical trials. (**A**) The Insightec ExAblate transcranial MR-guided FUS system. A hemispheric phased-array helmet (foreground) is coupled to the scalp via degassed circulating water and operated within an MRI bore, enabling real-time anatomic targeting, MR thermometry and post-treatment confirmation of blood–brain barrier opening on contrast-enhanced T1 imaging. (**B**) The NaviFUS neuronavigation-guided low-intensity FUS system, shown in a procedure room. The platform integrates a 256-element phased-array transducer (foreground) with optical neuronavigation tracking (Medtronic StealthStation, Medtronic Corp, Minneapolis, MN, USA, upper left), co-registered to the patient’s MRI, allowing treatments to be delivered outside an MRI environment. SonoCloud-9 is not depicted here, as it is a fully implanted intracranial device without an external form factor comparable to panels (**A**,**B**); see Table 1 for a full comparative summary of all three platforms, including guidance modality, invasiveness, skull attenuation, treatment paradigm, and clinical indications.

Three FUS technologies currently dominate active clinical trials for brain cancer: the Exablate (InSightec, Haifa, Israel), SonoCloud-9 (CarThera Corp, Lyon, France), and NaviFUS (NaviFUS Corp, Taipei, Taiwan) units (Figure 1 and Table 1) [2]. The Exablate device is a magnetic resonance imaging (MRI)-guided transcranial FUS system that uses a hemispheric phased-array helmet with 1024 elements and allows for real-time targeting and thermometry and for confirmation of blood–brain barrier (BBB) opening with contrast-enhanced MRI [3]. MRI is the most widely utilized modality for guiding and monitoring FUS brain therapies and is also used in SDT and liquid biopsy trials [3,4]. The SonoCloud-9 is an implantable nine-emitter device positioned directly into the craniotomy site at the time of tumor resection. By bypassing the skull-related acoustic attenuation challenges, the SonoCloud-9 enables the treatment of large-volumes of tumor and repeated outpatient BBB opening across multiple chemotherapy cycles [5]. NaviFUS is a noninvasive, neuronavigation-guided, low-intensity FUS system built around a 256-element phased-array transducer (with up to 32 elements used as receivers for passive acoustic imaging), allowing treatments to be delivered in a non-MRI based procedure room without a stereotactic frame. Additional brain FUS technologies from Alpheus Medical (Chanhassen, MN, USA), Delsona Therapeutics (New York, NY, USA), and Cordance Medical (Mountain View, CA, USA) are also under clinical evaluation.

### 2.3. FUS-Mediated Blood–Brain Barrier Opening for Drug Delivery

BBB opening is the most clinically mature application of FUS in neuro-oncology. Many drugs with established systemic anti-tumor activity have only modest central nervous system (CNS) penetration. FUS plus intravenously administered microbubbles transiently opens the BBB at the FUS focal spot for hours, providing spatial control over targeted drug delivery to the brain [2]. Mainprize and colleagues performed an early first-in-human MR-guided FUS BBB-opening study in primary brain tumors, with focal contrast enhancement that resolved within ~20 h and no significant adverse events [3]. Idbaih and colleagues subsequently showed in 19 patients with recurrent GBM that repeated SonoCloud-1 BBB disruption with carboplatin was safe, with a median overall survival of ~12.9 months in optimally treated patients [6]. Sonabend and colleagues evaluated repeated blood–brain barrier opening with the implantable SonoCloud-9 device in 17 patients with recurrent glioblastoma (GBM), administering albumin-bound paclitaxel every three weeks for up to six cycles. In a pharmacokinetic subgroup of seven patients, paired biopsies demonstrated a 3.7-fold increase in paclitaxel concentration in sonicated compared with nonsonicated peritumoral brain, providing the first direct human tissue quantification of ultrasound-enhanced drug delivery across the blood–brain barrier. In a separate three-patient carboplatin cohort, sonication increased the brain carboplatin concentrations 5.9-fold [5]. More recently, Woodworth and colleagues reported the multicenter BT008NA phase 1/2 trial of FUS plus adjuvant temozolomide in newly diagnosed high-grade glioma; in 34 evaluable participants, the median overall survival was 31.3 months versus 19.3 months in matched controls [7]. Beyond gliomas, Meng and colleagues showed that MR-guided FUS could enhance trastuzumab delivery to HER2-positive brain metastases, increasing radiolabeled antibody uptake by ~101% in targeted lesions versus –18% in non-targeted controls [8]. 

### 2.4. FUS-Enabled Liquid Biopsy

FUS-enabled liquid biopsy is a diagnostic extension of BBB opening since the same barrier disruption that allows drugs to penetrate into the CNS also releases tumor-derived cell-free DNA (cfDNA), extracellular vesicles and brain-specific proteins into peripheral blood. In a proof-of-concept study, Meng and colleagues performed serial MR-guided FUS in 9 patients with GBM; after sonication, plasma cfDNA, neuron-derived extracellular vesicles and S100B increased approximately 2.6-, 3.2-, and 1.4-fold, respectively [4]. In the one patient known to harbor the IDH1 tumor mutation, mutant copies were enriched two- to three-fold by droplet digital PCR [4].

### 2.5. Sonodynamic Therapy

Unlike BBB opening, SDT is intended as direct tumor-targeted therapy. Small-molecule sonosensitizers—most prominently 5-aminolevulinic acid (5-ALA) and its parenteral analogue SONALA-001—are converted intracellularly to protoporphyrin IX and accumulate preferentially in glioma cells; non-thermal FUS activates these molecules to generate cytotoxic reactive oxygen species within the tumor cells while largely sparing surrounding brain [9]. A first-in-human trial of 5-ALA SDT in recurrent high-grade glioma similarly demonstrated safety and tolerability with direct in vivo evidence of tumor-cell death [10].

### 2.6. Current State of the Field

**Figure 2:**
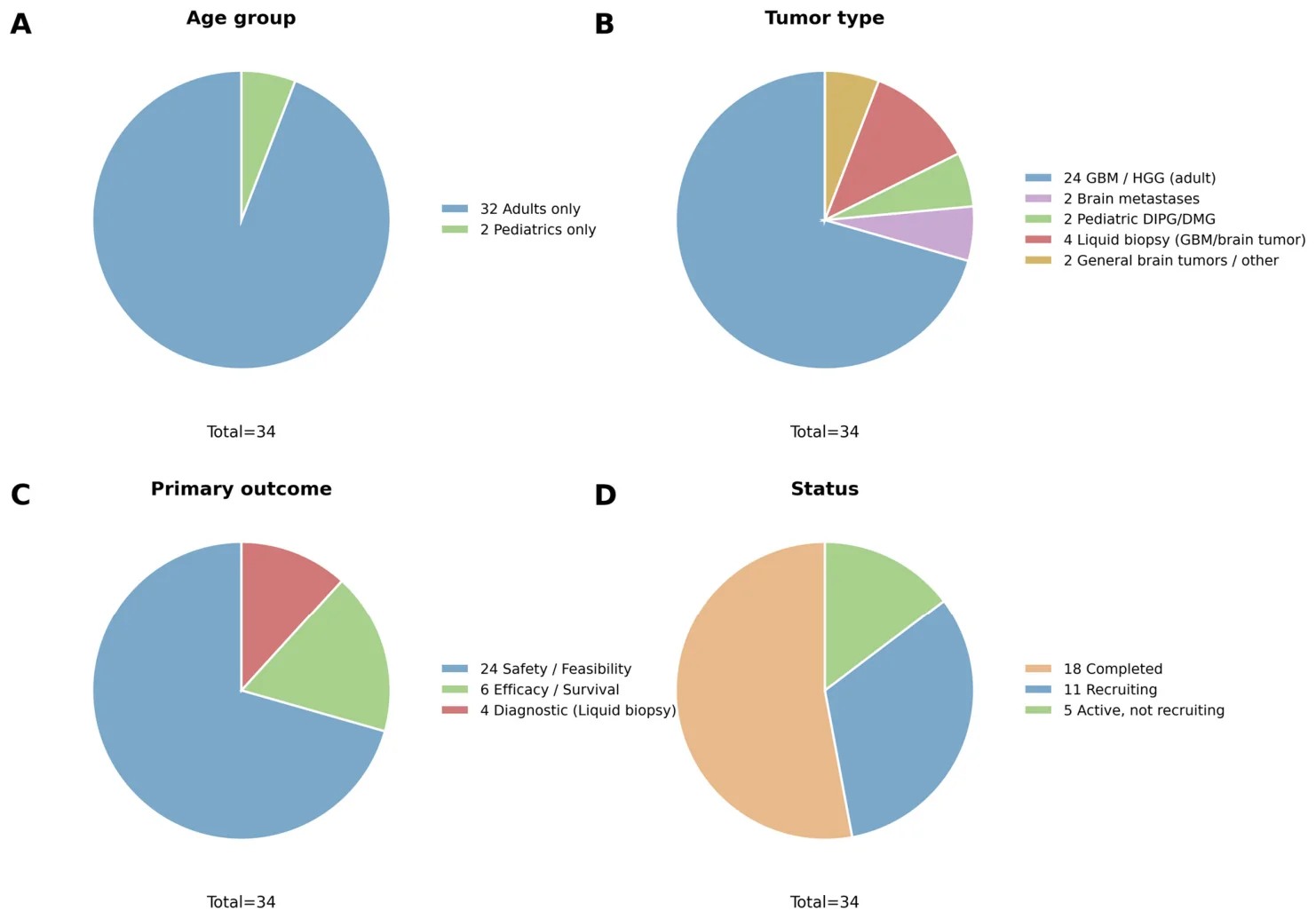
Trial status parameters across completed and ongoing focused-ultrasound (FUS) clinical trials for all brain cancer (*n* = 34 registered clinical trials) are summarized in these pie charts (*n* = 34) [data compiled from ClinicalTrials.gov, June 2026]. (**A**) Age group: the overwhelming majority of trials enroll adults only (*n* = 32), with two pediatric-only studies. (**B**) Tumor type: adult (newly diagnosed and recurrent) dominates the portfolio (*n* ≈ 24), followed by dedicated FUS-enabled liquid-biopsy studies (*n* = 4), brain metastases (*n* = 2), pediatric (*n* = 2), and general/other brain-tumor cohorts (*n* = 2). (**C**) Primary outcome: trials are predominantly safety/feasibility (*n* = 24), with a growing subset of efficacy/survival trials (*n* = 6) and dedicated diagnostic (liquid biopsy) trials (*n* = 4). (**D**) Status: completed trials (*n* = 18) and ongoing trials (*n* = 16; 11 recruiting and 5 active-not-recruiting) are roughly evenly distributed, suggesting that the evidence base is possibly shifting from feasibility-focused toward efficacy-focused endpoints. Abbreviations: GBM = glioblastoma; HGG = high-grade glioma; DIPG = diffuse intrinsic pontine glioma; DMG = diffuse midlineglioma. (Data from ClinicalTrials.gov, June 2026).

A large number of FUS clinical trials for brain cancer have been completed and have demonstrated favorable results and confirmation of the successful and safe use of FUS for BBB opening at targeted sites and in different grades of gliomas, with uptake of the specific antibody and/or drug into the target lesion and favorable responses of the lesions being treated. The literature search, conducted independently by the authors, identified the 34 trials summarized in Figure 2 and Table 2.

Representative examples of ongoing clinical trials are listed in Table 2.

### 2.7. Future Directions

FUS-enabled liquid biopsies and SDT are promising and emerging FUS applications. The critical next step is randomized confirmation that FUS improves clinical outcomes. Despite these advances in FUS technologies, challenges remain, including defining optimal treatment protocols; determining the timing, frequency, and integration of FUS with systemic therapies; and accounting for the variability in skull thickness and structure.

In summary, BBB opening is the most clinically mature FUS application in neuro-oncology, with multicenter phase 1 data confirming its safety across repeated cycles and direct tissue evidence of increased drug delivery, with a survival benefit in high-grade glioma being suggested in one non-randomized comparison study, but yet to be confirmed in a randomized trial. Sonodynamic therapy and FUS-enabled liquid biopsy remain at the safety/feasibility stage, supported only by small first-in-human studies. Continued enrollment in the active trials summarized in Table 2 will determine whether these early signals translate into confirmed efficacy.

## 3. Liver Tumors and Histotripsy

### 3.1. Methods and References Rationale

The following review of hepatic histotripsy includes the available published literature at the time of article submission. The authors performed a PubMed search to include manuscripts whose focus was on the evaluation and clinical implementation of “hepatic histotripsy.” Special attention was given to all in-human articles and was inclusive of all study types, regardless of the study design and the outcomes assessed. Given the paucity of published clinical outcomes and efficacy data to date with the application of histotripsy for the treatment of liver tumors, clinical conclusions drawn from this review and the current literature must be interpreted in this light.

### 3.2. Background

First introduced in 2004, histotripsy has taken the better part of the past two decades to mature into clinical relevance, as efforts have focused on creating a reliable and reproducible cavitation cloud that can be controlled in size and location [11,12,13]. Histotripsy uses high-intensity short-duration FUS pulses that cause naturally occurring microscopic gas bubbles in the targeted cells to expand and collapse, leading to cavitation effects, mechanical disruption, and liquefaction of the cells. It relies on mechanical forces rather than heat for tissue destruction, while sparing cellular proteins and antigens as well as structures rich in collagen like blood vessels and bile ducts [14]. Histotripsy (Histosonics Corp, Plymouth, MN, USA) received FDA approval for the treatment of liver tumors in the fall of 2023. After calibration and creation of a reliable cavitation cloud, the clinical treatment zone was successfully implemented with in vivo evaluations. The commercially available device utilizes a “bubble cloud” that measures about 3 × 3 × 6 mm, and the tumor is treated in a precessing helical fashion [15].

### 3.3. Current State of the Field

The first in-human THERESA trial and pivotal HOPE4LIVER study ultimately provided the data that lead to FDA-approval for histotripsy in the treatment of liver tumors [15,16]. Both trials demonstrated the ability to create focal tissue destruction within the liver, regardless of the tumor pathology or biology. The pivotal trial enrolled and treated 44 patients, meeting both primary endpoints of technical success (95.5%) as well as the predetermined safety performance goal. The tumor biology was approximately evenly split between hepatocellular carcinoma (HCC) and metastatic non-HCC, with the mean treated tumor size being 1.5 cm. Seven serious adverse events were identified during follow-up.

Subsequent multicenter studies have increased the understanding of histotripsy treatments for cancers involving the liver, expected clinical efficacy, and complications. Wehrle at al. retrospectively evaluated 510 tumors in 295 patients, from 18 different centers [17]. The average treated tumor size was 2.85 cm, with the plurality of tumors treated being of colorectal cancer origin. Only three severe complications were observed, all of which were disease progression. Additional reports have provided further technical and clinical outcomes data (Table 3). The only available survival data remain linked to the pivotal trial, demonstrating 74% and 49% 1-year survival for HCC and metastatic disease, respectively. The studies listed in Table 3 are quite variable in design and outcomes measured, as well as whether or not the liver tumors were partially or completely treated [17,18,19,20,21,22,23]. They do provide a general framework for discussion related to the clinical application of histotripsy for patients with liver cancer. However, given the heterogeneity in the survival outcomes data for these two patient populations (completely versus partially treated tumors) and dependence on the clinical oncologic stage at time of hepatic malignancy diagnosis, survival outcomes data cannot be fully assessed or compared in the patient populations in these articles [24,25].

Discrepancies have been observed in complication rates following hepatic histotripsy. Specifically, reports of cancer hyperprogression, thrombotic events, and hemorrhagic complications are appearing in the literature [17,26,27]. Breuer et al. reported a 3% rate of serious vascular injury, and there are other reports of an increase in the frequency of complications as compared to prior reports [26]. The frequency and types of adverse events require additional tracking and evaluation, as many of these newly observed adverse events were not noted in preclinical models [14,15,16,17,26,27,28].

### 3.4. Clinical Examples of Treatments

**Figure 3:**
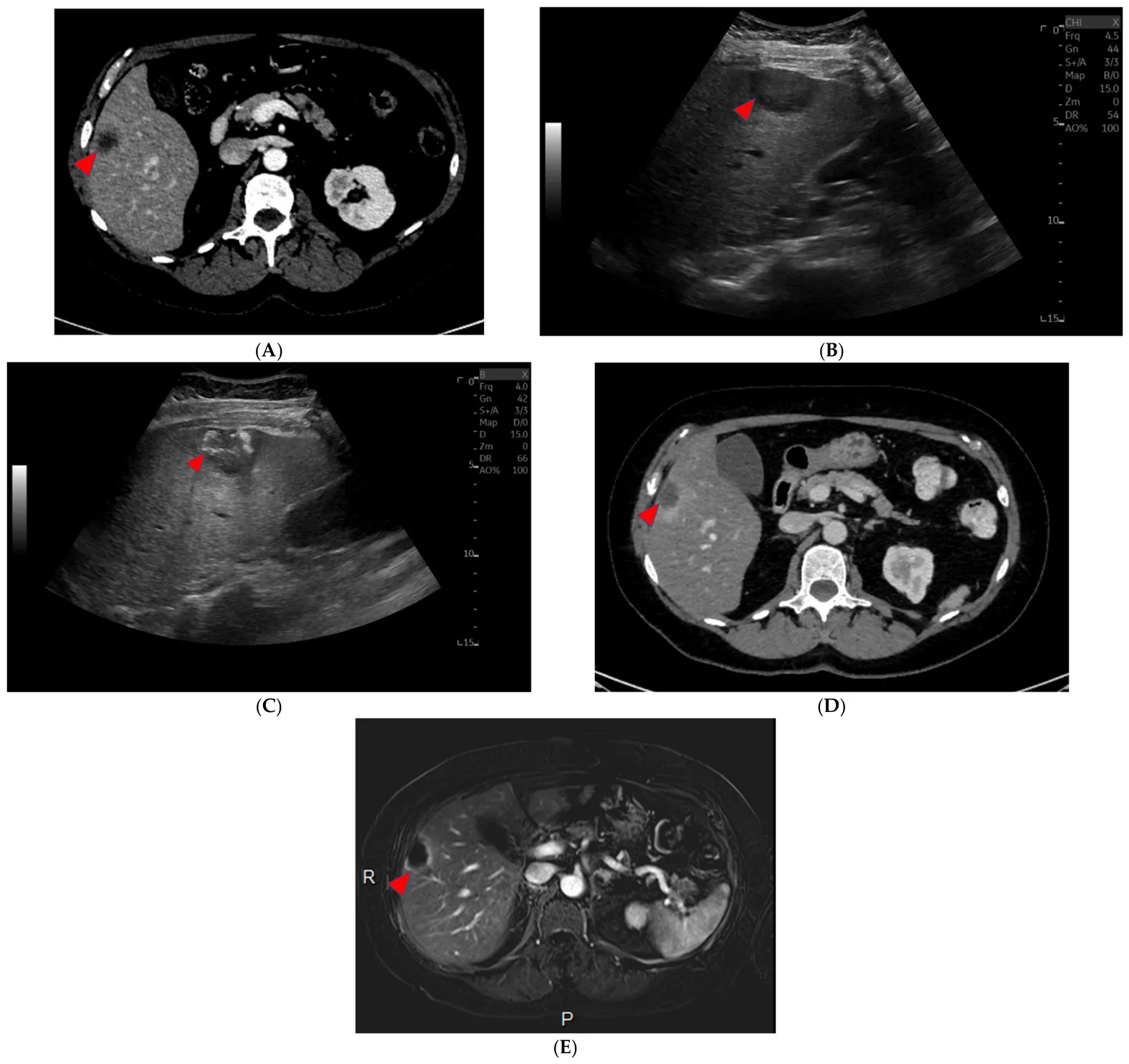
(**A**–**E**): Institutional review board approval was not required for this de-identified patient case. A 67-year-old female with history of oligometastatic breast cancer with a solitary liver metastasis (2.5 cm) that was enlarging despite treatment with systemic ribociclib was referred for histotripsy with a plan to initiate new systemic therapy post treatment. Per our standard work-up, the patient was evaluated for the tumor size, number and location and the potential for alternative therapy options. A screening ultrasound was performed in clinic to ensure the lesion could be treated with histotripsy. (**A**) Contrast-enhanced (CE) CT demonstrates a hypoenhancing solitary mass in hepatic segment 5 (arrowhead). (**B**) Planning preprocedural grayscale ultrasound image demonstrates a capsular-based hypoechoic mass (arrowhead) correlating to the tumor seen on CT imaging. (**C**) Immediate post-histotripsy grayscale ultrasound imaging demonstrates expected acoustic shadowing and echogenic foci of gas in the treatment volume (arrowhead). (**D**) One-month CE CT scan image demonstrates the rim-enhancing mass without complicating features (arrowhead). (**E**) Subtracted CE T1 weighted MRI demonstrates thin rim enhancing treatment zone (arrowhead), 2 months post-therapy. For image orientation, R = right side of patient and P = posterior/back of patient.

**Figure 4:**
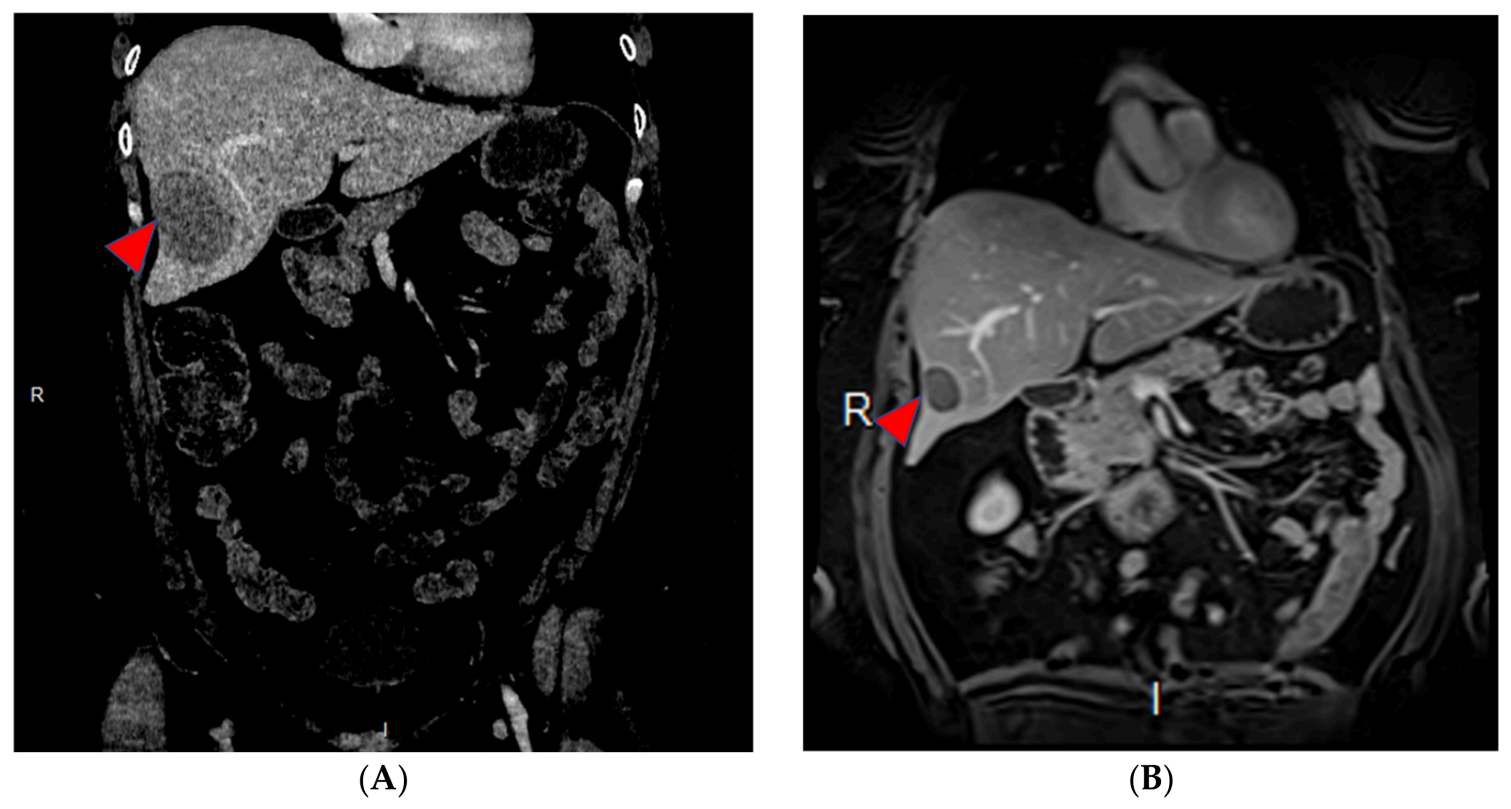
(**A**,**B**) Institutional review board approval was not required for this de-identified patient case. Patient with a metatstatic neuroendocrine tumor to segment 6 of the liver treated with histotripsy. (**A**) Metastatic neuroendocrine tumor in segment 6 (arrow) pre-histotripsy, coronal reconstruction. (**B**) Ten-month follow-up MRI with contrast showing interval decrease in size of the segment 6 mass (arrow), coronal reconstruction. Image orientation: R = right side of patient.

Histotripsy is now another tool in the armamentarium of locoregional therapies (LRT) for both primary and secondary liver cancer. (Figure 3 and Figure 4) The nuances of patient selection and the appropriateness of when to treat and when not to treat with histotripsy remain to be better defined, as clinicians seek to better understand the risks, benefits, and clinical outcomes for particular patient scenarios. The evidence has thus far focused on safety, technical success and imaging response to histotripsy. However, as with other LRT options, prospective survival outcomes are needed to better enable appropriate implementation of this promising technology [29,30,31].

Using general anesthesia, paralytics, and tightly controlled ventilatory tidal volumes, histotripsy was performed. The patient was discharged on post-procedure day 1 without any immediate complications. Laboratory values one-week post-treatment showed new asymptomatic transaminitis (AST 623 U/L and ALT 276 U/L) that normalized by 6 weeks. The patient experienced mild muscular soreness at the beam entry site lasting two weeks. Follow-up imaging demonstrated appropriate targeting of the tumor, without complicating features, at both one and 2 months.

### 3.5. Future Directions

There remains much interest in the future applications of histotripsy for the treatment of cancers involving the liver. Preclinical work continues to improve targeting beyond grayscale and color Doppler ultrasonography, such as with the use of contrast-enhanced ultrasound and/or multimodal fusion imaging [32,33]. In addition, the prospect of an abscopal effect following histotripsy, while promising, remains in preclinical evaluation [34,35,36].

In summary, hepatic histotripsy is a promising new therapeutic option for patients with both primary and metastatic liver cancer, but the use of this technology in this clinical scenario will benefit from additional and more robust validation. Treatment paradigms and clinical appropriateness continue to be studied and require further multidisciplinary evaluation [29]. While noninvasive, both immediate adverse events and long-term outcomes are pending additional study. Likewise, the expansion of eligible patients for histotripsy and its secondary therapeutic benefits remain to be better understood and codified [37].

## 4. Prostate Cancer

### 4.1. Methods and References Rationale

A literature search was conducted using PubMed/MEDLINE, and Google Scholar in August 2025. These databases were selected for their complementary coverage. We also queried EU Clinical Trials Register to verify FDA approval status and ongoing trial data for HIFU devices. Search terms combined controlled vocabulary (MeSH: High-Intensity Focused Ultrasound Ablation, Prostatic Neoplasms/therapy, Treatment Outcome, Patient Selection) with free-text Boolean queries: (HIFU OR “high-intensity focused ultrasound”) AND “prostate cancer” AND (“whole gland ablation” OR “focal therapy” OR “partial gland ablation”) AND (outcomes OR “clinical trial” OR “radical prostatectomy”). Exclusion criteria comprised case reports, editorials, animal studies, purely technical engineering reports, and superseded publications from duplicate cohorts.

This search strategy carries several inherent limitations. First, restricting the primary analysis to English-language publications may have introduced language bias, potentially excluding relevant data from non-English-language centers with substantial HIFU experience. Second, the heterogeneity in study designs across the included literature limits the direct comparability of outcomes in the absence of randomization. Third, the absence of a formal systematic review protocol or PRISMA-compliant screening process could potentially lead to reviewer bias. Moreover, publication bias remains a concern, as studies reporting favorable HIFU outcomes are more likely to be published and indexed, potentially overrepresenting efficacy data relative to real-world complication and failure rates.

High-intensity focused ultrasound (HIFU) has emerged as a minimally invasive therapeutic option for localized prostate cancer. HIFU delivery involves precise anatomical planning using multiparametric MRI and real-time ultrasound guidance, with transrectal ultrasonography enabling targeted thermal ablation of cancerous tissue while preserving surrounding structures [38]. The evolution of HIFU technology has prompted extensive clinical investigation into its oncological efficacy and functional outcomes, particularly in comparison to established techniques such as radical prostatectomies (RP).

### 4.2. Whole-Gland Ablation Trial Results

Clinical trials evaluating whole-gland HIFU ablation for prostate cancer have demonstrated promising medium-term outcomes, though with variable biochemical control rates that are dependent on risk stratification and follow-up duration. Table 4 summarizes the key landmark trials for whole-gland HIFU treatment.

The landmark HIFI trial, a prospective nationwide non-inferiority study comparing HIFU to RP in 3328 patients reported a 30-month salvage treatment-free survival rate of 89.8% for HIFU versus 86.2% for RP, with no distant metastases or prostate cancer-specific deaths in either group [39]. This study notably demonstrated the non-inferiority of HIFU for cancer control while achieving superior functional preservation. However, the long-term comparative data reveal more nuanced outcomes. Rosenhammer et al. reported 10-year results comparing whole-gland HIFU to open RP, showing cancer-specific survival of 94% for HIFU versus 98% for RP, with a biochemical-free survival of 70% and 80%, respectively [40]. Importantly, HIFU demonstrated significantly reduced cancer-specific survival in intermediate-risk (*p* = 0.010) and high-risk (*p* = 0.048) patients, suggesting that patient selection is critical for optimal outcomes [40].

### 4.3. Partial and Focal Gland Ablation Trial Results

Focal and partial-gland HIFU ablation strategies have gained considerable traction as tissue-sparing alternatives, with accumulating evidence supporting their oncological viability in selected patients. Table 5 presents the key focal and partial ablation prostate cancer trial outcomes.

The PART feasibility randomized controlled trial compared HIFU partial ablation (primarily hemiablation) to RP in intermediate-risk prostate cancer patients, demonstrating the feasibility of randomization and did report the biochemical disease-free rates of 78–84% at 1 year for HIFU, with superior urinary and sexual function preservation compared to radical prostatectomy [43]. The largest multi-institutional cohort study to date, analyzing 1379 men treated with focal HIFU over 15 years demonstrated a 7-year failure-free survival of 69%, with 68% for intermediate-risk and 65% for high-risk cancers [44]. Remarkably, this study reported 7-year metastasis-free and prostate cancer-specific survival of 100%, with salvage whole-gland or systemic treatment-free survival of 75% at 7 years [44].

### 4.4. Comparison with Standard Whole-Gland Therapies

Direct comparative studies between HIFU and standard radical surgical therapies have consistently demonstrated HIFU’s superior functional outcomes, although some trade-offs in oncological control were noted depending on patient selection. Table 6 presents the comparative outcomes between HIFU and RP.

The HIFI trial showed that HIFU resulted in significantly better urinary continence (relative risk 0.76, *p* < 0.001) and erectile function preservation, with a median IIEF-5 score decrease of only 4 points compared to 9 points after radical prostatectomy (*p* < 0.001) [39]. The propensity-matched analysis by Shah et al. demonstrated that focal therapy achieved a failure-free survival of 92%, 89%, and 86% at 3, 5, and 8 years respectively, versus 86%, 82%, and 79% for laparoscopic radical prostatectomy (*p* = 0.03), while maintaining pad-free continence in 97% versus 86% (*p* = 0.0007) and erectile function sufficient for penetrative sex in 68% versus 39% (*p* < 0.0005) [49].

### 4.5. FALCON Consensus on Patient Selection

In current clinical practice, the role of HIFU is strictly for the focal ablation of prostate cancer. The FALCON (Focal therapy: Ablation of Localized Cancer of the prostate) consensus guidelines and emerging evidence strongly supports rigorous patient selection criteria for optimal HIFU outcomes. Table 7 summarizes the patient selection criteria derived from the contemporary evidence.

Patient selection is crucial for focal therapy success, requiring systematic risk assessment, refined stratification and a solid diagnostic approach using transperineal template saturation biopsies combined with MRI/TRUS fusion-targeted biopsies for PI-RADS ≥ 3 lesions [51]. Contemporary selection criteria for focal HIFU typically include low-to-intermediate-risk localized prostate cancer with MRI-visible lesions, PSA ≤ 15 ng/mL, ISUP Grade Group ≤ 3, and ≤2 suspicious lesions on multiparametric MRI [47]. Critical exclusion criteria include tumors abutting the urinary sphincter or urethra, high-volume Gleason 6 or any Gleason ≥ 7 disease in untreated areas and bilateral disease requiring neurovascular bundle ablation as well as clinically advanced stage (≥T3a) [44]. 

### 4.6. Technical Limitations

Body habitus limitations include rectal stenosis or an inability to accommodate a transrectal probe. Prostate volume > 40 mL is generally acceptable. Extensive prostatic calcifications also impede ultrasound transmission. Anterior tumors and apical lesions near the external sphincter pose technical challenges, with apical lesions risking sphincter injury and incontinence [46].

### 4.7. Complications

Acute urinary retention occurs in 3–10% of patients after FUS treatments, typically managed with temporary catheterization [46]. Rectourethral fistula is rare (<1%) but serious. Erectile dysfunction affects 20–25% of preoperatively potent patients, significantly lower than 60–70% seen after RP [41,45]. Urinary incontinence is uncommon (2–6%) and mostly transient [46]. Other complications also seen include urinary tract infection (5–10%), urethral stricture (3–5%), and lower urinary tract symptoms [46].

### 4.8. Future Directions

Ongoing registries will accumulate long-term outcomes data beyond 10 years. Combination strategies with immunotherapy are under investigation. Artificial intelligence-guided treatment planning using deep learning algorithms for automated lesion segmentation and ablation zone optimization shows promise in improving precision and reducing operator variability.

In summary, focal HIFU represents a minimally invasive treatment for localized prostate cancer with superior functional preservation compared to RP. At this point, whole gland HIFU is considered investigational especially for higher risk prostate cancer. However, in well selected patients, focal HIFU provides comparable oncological outcomes with superior functional outcomes compared to RP. Complications related to the use of FUS for prostate cancer include urinary retention (3–10%), erectile dysfunction (20–25%), and rare fistula formation (<1%). Future directions with FUS in the treatment of prostate cancer include biomarker development to predict and/or assess outcomes, AI-guided treatment planning, and its use in combination with immunotherapy.

## 5. Breast Cancer

### 5.1. Methods and References Rationale

This section provides a focused narrative synthesis of the breast cancer (BC) literature rather than a formal systematic review. Relevant studies were identified by searching PubMed and ClinicalTrials.gov through June 2026, using combinations of the terms focused ultrasound, histotripsy, microbubble, and breast cancer. We prioritized completed and ongoing clinical trials, supplemented by seminal early-phase studies, and a limited number of preclinical reports. Clinical studies were included when they directly evaluated an FUS intervention in patients with primary or metastatic BC and reported safety, feasibility, pathology, imaging, immune, or local-control outcomes. Duplicate reports were consolidated by the NCT identifier. BC was included because of its superficial anatomy and clinical experience spanning different modalities. Several limitations to this review should be acknowledged. A pre-registered protocol and structured risk-of-bias assessment were not used, and selection or reporting bias in the cited literature therefore cannot be excluded. The available breast FUS literature is heterogeneous in design, endpoints, and follow-up, limiting direct comparisons and quantitative analyses. Accordingly, the summaries below are intended to outline the current clinical landscape rather than provide a definitive evaluation or a meta-analysis, for which a dedicated manuscript on this topic would be warranted. 

Breast cancer (BC) remains the most commonly diagnosed cancer and the second-leading cause of cancer-related death in women worldwide [53]. Optimal BC treatment centers use a multidisciplinary strategy integrating surgery, systemic therapy, and radiation, with each carrying its own risk. FUS is being explored as a noninvasive approach to local BC treatment and as a platform for combination strategies that may enhance systemic therapies, with early clinical studies supporting its feasibility and favorable safety profile.

Primary BC is an attractive target for FUS given its relatively superficial anatomic location. There is precedent for omitting surgical resection in favor of ablative treatment for select breast cancers. In 2025, the FDA granted de novo marketing authorization for cryotherapy in low-risk, early-stage BC [54]. FUS ablation holds several advantages over cryotherapy. It is noninvasive, requires no skin incision or probe insertion, and allows for precise, repositionable treatment focal zones. Steep thermal gradients produce sharply demarcated areas of coagulative necrosis with minimal surrounding tissue injury. Real-time imaging guidance further enables accurate tumor targeting and confirmation of the treatment effect.

### 5.2. Thermal Ablation

Several early clinical trials have explored the feasibility and safety of HIFU ablation for primary BC (Table 8). These early trials are predominantly single-arm or non-randomized with limited follow-up and are best interpreted as early evidence. Wu et al. randomized 48 patients with T1–T2 disease to modified radical mastectomy with or without neoadjuvant HIFU and reported complete coagulative necrosis with no severe adverse events. Immunohistochemical analysis suggested that tumor cells lost their proliferative and metastatic potential [55]. A subsequent series by Wu et al. in 22 patients with larger tumors (mean tumor diameter = 3.4 cm) reported complete tumor necrosis at biopsy, with progressive replacement by fibroglandular tissue and complete fibrosis by 6–12 months [56]. MR-guided FUS (MRgFUS) trials by Furusawa et al. in 30 and 21 patients, respectively, reported the ablation of ≥95% of tumor volume in 90% of cases and complete necrosis in 95% of patients, with the single local recurrence reliably identified on contrast-enhanced MRI [57,58]. Notably, these HIFU trials enrolled younger patients with larger tumors than those included in cryotherapy trials, underscoring the potential applicability of FUS across a broader BC population. Across these series, however, the outcomes are limited to pathological necrosis and imaging response, with comparator arms and long-term oncologic endpoints generally absent, thereby making selection bias difficult to exclude. The technology will therefore require validation in larger controlled studies. 

### 5.3. Histotripsy

Following FDA de novo clearance of histotripsy for hepatic tumors in 2023, interest has grown in extending this non-thermal mechanical ablation technique to other solid malignancies. A recent case report demonstrated a complete metabolic response after staged histotripsy of multifocal liver metastases from ER-positive breast cancer [62]. However, direct application of histotripsy to primary breast tumors in humans has not been reported, and future clinical trials are anticipated.

### 5.4. Immunomodulation and Combination Therapy

Beyond direct ablation, FUS has the potential to function as an immune adjuvant. In the Wu et al. randomized trial, patients in the HIFU arm demonstrated upregulation of HSP70 and increased circulating CD3+, CD4+, CD8+, B lymphocytes, and NK cells compared with surgery alone, with concordant immune activation in tumor-draining lymph nodes [55]. These single-study findings suggest an immunostimulatory effect and provide the rationale for the combination trials below. A two-arm pilot study in metastatic BC (Breast 48, NCT03237572) evaluated the subtotal HIFU ablation of a single lesion combined with pembrolizumab (PD-1 blockade), randomizing patients to receive immunotherapy before or after HIFU to assess how sequencing modulates local and systemic immune activation. Accrual is complete and immune profiling analyses are ongoing [63]. An actively enrolling Phase I trial (Breast 54, NCT04796220) combines thermally ablative FUS with low-dose gemcitabine in stage I–III BC, building on preclinical data showing that this combination may improve tumor control in an adaptive immunity-dependent manner [64].

FUS has also been explored as a radio enhancement strategy. In a clinical trial of 21 patients (NCT04431674), MRgFUS combined with microbubbles (MRgFUS-MB) was employed to increase the tumor sensitivity to ionizing radiation. No grade ≥ 3 microbubble-related adverse events occurred, 83% of tumors achieved partial or complete response, and the 2-year local control rate was 76% (95% CI 54–100%) [65]. No devices are currently FDA-approved for these applications. Table 9 summarizes open and recently completed clinical trials.

### 5.5. Limitations

Anatomic constraints influence patient selection. An inadequate distance between the tumor and critical structures (skin or chest wall) may preclude safe acoustic delivery. Achieving a reliable transducer seal around the nipple–areolar complex or in the axillary region can be technically challenging, though devices that submerge the breast in a water bath or can create better contact with the skin may mitigate these challenges. A fundamental limitation shared with all ablative strategies is the absence of a surgical specimen for histopathologic margin assessment.

### 5.6. Follow-Up and Surveillance

Establishing post-treatment surveillance imaging protocols is essential. Contrast-enhanced MR and mammographic imaging are utilized post-ablation, with the absence of enhancement within the treated zone indicating successful ablation. The development of new or persistent areas of enhancement at follow-up warrants a biopsy.

### 5.7. Risks and Complications

Published FUS ablation trials in BC report low rates of serious adverse events. Skin burns at the acoustic entry site represent the most commonly reported adverse event and are generally manageable with conservative wound care. Rib periostitis or pain may occur when tumors are near the chest wall. Device-specific complications, such as microbubble-related events in MRgFUS-MB protocols, have been mild in reported series. No procedure-related deaths have been reported in the published BC FUS literature.

### 5.8. Future Directions

The landscape for FUS in BC is evolving rapidly. Ongoing trials will define the optimal role for FUS as monotherapy or in combination with immunotherapy, chemotherapy and/or radiation. Advances in real-time MR thermometry, treatment planning software, and artificial intelligence technologies will continue to improve targeting precision and treatment delivery. Regulatory approval of a FUS device for BC treatment would represent a meaningful advance for patients seeking minimally invasive therapeutic options.

In summary, FUS remains investigational but is a promising fully noninvasive approach to local BC treatment, with the emerging potential to be used alongside current systemic therapies. Thermal ablation is the most clinically mature application to date, with early studies supporting favorable short-term safety, pathological necrosis, and imaging response in appropriately selected patients. However, long-term comparative data on local control and survival are limited. FUS-based immunomodulation and combination approaches with checkpoint inhibition, chemotherapy, and radiation are early in development and supported by initial immunostimulatory findings and several ongoing trials. Histotripsy for primary BC remains at a preclinical stage. Continued prospective investigation, including larger and when feasible, comparative trials with standardized endpoints and long-term follow-up will be important to define the role of FUS in multidisciplinary BC care. 

## 6. Conclusions

Advancements in FUS technologies provide an exciting convergence of imaging guidance with precisely targeted delivery of acoustic energy to potentially treat cancer noninvasively. Depending on the acoustic parameters used, FUS can function as a thermal ablation tool, a mechanical disruptor, a drug-delivery enhancer, or an immunologic modulator. The versatility of FUS enables its integration with a wide range of existing therapies, including chemotherapy, immunotherapy, other biologics, and radiation. Indeed, above and beyond the solid tumors and device platforms described in this review article, FUS is also being used and showing promising results in ongoing clinical trials using some of the technologies described above and other device platforms not included in this review for the treatment of cancers and tumors involving the pancreas, kidney, musculoskeletal system, soft tissues, skin and thyroid gland with and/or without an associated immunotherapeutic agent. 

Despite the significant potential of FUS technologies being able to advance treatment paradigms of solid tumors and cancers, several barriers remain related to its translation into the clinical arena. These points of friction include the need for better standardization of the treatment protocols, variability in device technology, certain technologies designed to treat single types and locations of cancers and limited long-term clinical and efficacy outcomes data. Cost and infrastructure requirements may also limit accessibility to this technology on a wider scale.

Future directions include integrating artificial intelligence into treatment planning, developing better real-time monitoring tools, and expanding the indications, as well as FDA-approval for use of FUS in other tumor types and organ systems. Continued research and robust clinical trial outcomes data will be essential to further define the role and impact of FUS technologies in cancer care. As clinical experience with FUS grows and technological innovations continue to advance, FUS has the potential to become an integral component of multidisciplinary and multidimensional cancer care in the future.

## Figures and Tables

**Table 1:** Comparative considerations for FUS devices in clinical brain cancer trials.

Domain	Insightec Exablate	CarThera SonoCloud-9	NaviFUS
**Guidance modality**	MRI-guided (real-time anatomic targeting, thermometry, and contrast-enhanced confirmation of BBB opening)	Implanted ultrasound emitter, guidance is built into the device position established at the time of cranial-window placement	Frameless neuronavigation co-registered with the patient’s MRI
**Invasiveness**	Noninvasive, transcranial	Minimally invasive, requires surgical implantation of a 9-emitter device in a cranial window	Noninvasive, transcranial
**Skull attenuation**	Attenuated by intact skull; mitigated by phased-array correction within the MR-bore helmet	Bypasses skull because emitters sit beneath the bone flap	Attenuated by intact skull; partially mitigated by neuronavigation-guided targeting
**Treatment paradigm**	Intermittent MRI-suite sessions	Repeated outpatient BBB opening sessions (every 2–3 weeks for up to 6+ cycles)	Workflow-flexible, repeated treatments deliverable outside the MRI suite
**Intra-treatment monitoring**	Real-time MR thermometry and acoustic-emissions feedback	Acoustic-emissions feedback; no intra-treatment MR thermometry	Acoustic-emissions feedback; no intra-treatment MR thermometry
**Sonication volume/scalability**	Focal spots tightly defined by transducer geometry; limited per-session coverage	Large peritumoral volumes via 9-emitter array; designed for repeated treatment	Mobile transducer; volume scaled by number of sonications per session
**Therapeutic indications under investigation**	BBB opening, FUS-enabled liquid biopsy, sonodynamic therapy (SDT)	Primarily BBB opening for drug delivery	BBB opening and SDT

Abbreviations: BBB = blood–brain barrier; FUS = focused ultrasound; MR(I) = magnetic resonance (imaging); SDT = sonodynamic therapy.

**Table 2:** Representative active focused-ultrasound clinical trials in brain cancer (data from ClinicalTrials.gov, June 2026).

Trial/Sponsor	NCT	Device	Phase	Tumor/Setting	FUS Use + Agent
***BBB opening: drug and immunotherapy delivery***					
SONOBIRD	NCT05902169	SonoCloud-9	3	Recurrent GBM	BBBO + carboplatin vs. SOC
LIMITLESS	NCT05317858	Exablate Neuro	3	NSCLC brain metastases	BBBO + immune checkpoint inhibitor
NaviFUS pivotal	NCT06496971	NaviFUS	3	Recurrent GBM	BBBO + bevacizumab vs. bevacizumab
Northwestern	NCT05864534	SonoCloud-9	2	Newly dx GBM	BBBO + botensilimab/balstilimab + liposomal doxorubicin
UVA (EGFR BATs)	NCT07343986	LIFU	1	MGMT-unmeth. GBM	BBBO + EGFR bispecific-armed T cells
InSightec	NCT05630209	Exablate Neuro	1/2	Pediatric DIPG	BBBO + doxorubicin
Columbia	NCT05762419	Neuronavigated FUS	1	Pediatric DMG	BBBO + oral etoposide
***Sonodynamic therapy***					
Alpheus	NCT07225621	Alpheus LIDU	2	Newly dx GBM	5-ALA SDT + temozolomide (randomized)
UVA	NCT06039709	LIFU	1	Recurrent GBM	5-ALA SDT
Mayo	NCT07076472	Exablate Neuro	Early 1	Recurrent GBM	SONALA-001 (IV 5-ALA) SDT
***FUS-enabled liquid biopsy***					
WashU (sonobiopsy)	NCT05281731	FUS sonobiopsy	Feasibility	Glioblastoma	cfDNA/biomarker release
UHN Toronto	NCT04940507	Exablate (MRgFUS)	Feasibility	Brain tumors	Circulating biomarker release

Abbreviations: Bolded and italicized words denote a subheading under which specific institutions and/or companies are listed; 5-ALA = 5-aminolevulinic acid; cfDNA = cell-free DNA; EGFR BATs = epidermal growth factor receptor bispecific antibody–armed T cells; FUS = focused ultrasound; IV = intravenous; LIDU = low-intensity diffuse ultrasound; LIFU = low-intensity focused ultrasound; MGMT = O^6^-methylguanine–DNA methyltransferase; MRgFUS = magnetic resonance–guided focused ultrasound; NCT = ClinicalTrials.gov identifier; NSCLC = non–small cell lung cancer; SOC = standard of care; SONALA-001 = parenteral (intravenous) aminolevulinic acid hydrochloride. Note: There is substantial heterogeneity among these trials in study design, phase, endpoints, follow-up duration, and treatment protocols. This table is included to provide a compendium of currently registered and active FUS clinical trials in brain cancer, not to directly compare each trial’s specific design or endpoints, but rather to convey the overall scope and maturity of the current trial landscape. For further details on any individual trial, please refer to the corresponding ClinicalTrials.gov identifier (NCT number) provided.

**Table 3:** Available published reports on the technical and clinical success of histotripsy for liver tumors. Note that patient populations and defined efficacies and outcomes are heterogeneous across these articles. Table 3 is included to provide an overview of currently available clinical trials data to date, with further details and analyses available through direct access of the compiled works cited.

Study	Analysis	Patient # (Tumor #)	Tumor Size	Immediate Technical Success	Treatment Efficacy	Major Complications per Patient
Wehrle et al. (2025) [17]	Retrospective	295 (510)	2.85 cm	N/A	N/A	1.3% (@ 30 days in 230 patients)
Wehrle et al. (2025) [18]	Retrospective	47 (91)	2.55 cm	N/A	95% (1 month)	0%
Ziemlewicz et al. (2025) [19]	Prospective	47 (52)	1.3 cm	95.7%	96% (1 year, post hoc)	13%
Mabud et al. (2025) [20]	Retrospective	21(39)	2.65 cm	100%	100% (1 month, 16 fully treated) *	0%
Chan et al. (2026) [21]	Prospective	30 (60)	1.8 cm	95%	82.5% (1 month)	0%
Liu et al. (2026) [22]	Retrospective	27 (35)	N/A	69% **	N/A	14.8%
Jimenez-Soto et al. (2026) [23]	Retrospective	27 (42) ***	3.0 cm	N/A	75% (6 of 8 patients @1 month) ***	11%

Abbreviations: N/A = not available. # = number. * = 23 of 39 tumors intentionally only partially treated. ** = 19 of 35 tumors intentionally only partially treated. *** = 12 of 42 tumors intentionally only partially treated. Note: Due to the marked heterogeneity and variability in the presented information analyzed in these cited studies, the authors of this review article have evaluated the available data (which were lacking in some of the manuscripts) in an effort to categorize treatment efficacy based on a per lesion assessment, which was complicated by the fact that some of the tumors were intentionally only partially treated, and to determine a major complication rate on a per patient basis rather than a per lesion basis.

**Table 4:** Landmark whole-gland HIFU ablation trials.

Trial/Study	Design	Sample Size	Follow-Up	Primary Oncologic Outcome	Functional Outcomes	Reference
HIFI Trial	Prospective non-inferiority study (HIFU vs. RP)	3328 total (1967 HIFU; 1361 RP)	Median 30 months	30-month STFS: 89.8% (HIFU) vs. 86.2% (RP); HR 0.76 (*p* = 0.008); No metastases or PC deaths	Continence better with HIFU (RR 0.76, *p* < 0.001); IIEF-5 decline: −4 (HIFU) vs. −9 (RP), *p* < 0.001	[39]
Rosenhammer et al.	Retrospective comparative (whole-gland HIFU vs. open RP)	164 HIFU; 328 RP	Median 6.5 years (HIFU); 13.3 years (RP)	10-year CSS: 94% (HIFU) vs. 98% (RP); 10-year BFS: 70% (HIFU) vs. 80% (RP)	Not primary focus	[40]
Nam et al.	Propensity-matched (RARP vs. whole-gland HIFU)	193 HIFU; 760 RARP	Up to 24 months	No difference in treatment-free survival	IIEF-5 at 6/12/24 mo: 9.5 vs. 4.8/9.5 vs. 5.8/8.4 vs. 6.7 (all *p* < 0.001 favoring HIFU); continence >96% both groups	[41]
Misraï et al.	Retrospective single-center	227 patients	Median 3.2 years	5-year biochemical-free survival: 30% overall; efficient control in low-risk only	Not reported	[42]

Abbreviations: HIFU = high-intensity focused ultrasound, STFS = salvage therapy-free survival; PC = prostate cancer; RR = relative risk; IIEF = international index of erectile function; RARP = robot-assisted radical prostatectomy.

**Table 5:** Landmark focal and partial-gland HIFU ablation trials.

Trial/Study	Design	Sample Size	Follow-Up	Oncologic Outcomes	Functional Outcomes	Reference
PART Trial *	Feasibility RCT (partial ablation vs. RP)	82 randomized (41 HIFU; 41 RP)	36 months planned	Biochemical disease-free: 78–84% at 1 year; 45–84% at 5 years (HIFU)	Superior urinary and sexual function vs. RP	[43]
Reddy et al. Multi-institutional	Retrospective cohort	1379 men	Median 50 months	7-year FFS: 69% overall; 68% (intermediate-risk); 65% (high-risk); 7-year MFS and CSS: 100%	Not primary focus	[44]
Van Velthoven Hemiablation	Prospective single-arm	50 patients	Median 39.5 months	5-year MFS: 93%; CSS: 100%; OS: 87%; BCR: 28–36%	Complete continence: 94%; erection sufficient for intercourse: 80%	[45]
Van Velthoven Phase IIa	Prospective phase IIa	20 patients	Median 38 months	BCR-free survival: 100% (1 yr), 89% (2 yr), 82.7% (3 yr); CSS and OS: 100%	Continence preserved in majority; erectile function maintained	[46]
Mala et al. Real-world	Retrospective cohort	57 men	Median 27.5 months	Positive biopsy: 33.3%; mpMRI suspicious: 29.8%; PSA decreased from 7.3 to 2.5 ng/mL	IIEF median: 16 → 11.5 (*p* < 0.001); complications: 19.3%	[47]

Abbreviations: MFS = metastasis-free survival; CSS = cancer-specific survival; OS = overall survival; BCR = biochemical recurrence; PSA = prostate-specific antigen; mpMRI = multiparametric magnetic resonance imaging. * This trial was structured to determine whether or not it was feasible to randomize patients between radical prostatectomy and HIFU. Even though the data from this trial are publicly available, the trial was not powered to show non-inferiority.

**Table 6:** HIFU versus radical prostatectomy—comparative outcomes.

Study	Design	Sample Size	Follow-Up	Oncologic Outcomes	Functional Outcomes	Reference
HIFI Trial	Prospective (HIFU vs. RP)	3328 (1967 HIFU; 1361 RP)	Median 30 months	STFS: 89.8% (HIFU) vs. 86.2% (RP); HR 0.76, *p* = 0.008	Continence RR 0.76 (*p* < 0.001); IIEF-5 Δ: −4 (HIFU) vs. −9 (RP), *p* < 0.001	[38]
Nyk et al.	Propensity-matched (focal HIFU vs. laparoscopic RP)	164 (82 HIFU; 82 RP)	12 months	Increased treatment failure risk with HIFU	Incontinence ATE: −0.808 (*p* < 0.01); ED ATE: 5.092 (*p* < 0.01), favoring HIFU	[48]
Shah et al.	Propensity-matched (focal therapy vs. laparoscopic RP)	492 (246 FT; 246 RP)	Median 49 months (FT); 64 months (RP)	FFS at 3/5/8 years: 92%/89%/86% (FT) vs. 86%/82%/79% (RP), *p* = 0.03	Pad-free: 97% (FT) vs. 86% (RP), *p* = 0.0007; erections for sex: 68% (FT) vs. 39% (RP), *p* < 0.0005	[49]
Anceschi et al.	Prospective cohort (HIFU vs. cryoablation vs. RARP)	196 total	24 months	Time to failure longer for RARP (*p* < 0.001)	Earlier ED recovery with HIFU; comparable continence and potency at follow-up; HIFU complications: 20.4% vs. RP: 2.75%	[50]

Abbreviations: ATE = average treatment effect; FT = focal therapy; FFS = failure-free survival; ED = erectile dysfunction.

**Table 7:** Patient selection criteria for HIFU therapy.

Selection Domain	Inclusion Criteria	Exclusion Criteria	Reference
Risk Stratification	Low-to-intermediate-risk localized PCa; Gleason ≤ 7 (ISUP Grade Group ≤ 2); PSA ≤ 15–20 ng/mL	High-risk disease; Gleason ≥ 8; High-volume Gleason 6 or any Gleason ≥ 7 in untreated areas	[51,52]
Imaging	MRI-visible lesions; PI-RADS ≥ 3; ≤2 suspicious lesions on mpMRI; Unilateral disease preferred	Bilateral disease requiring NVB ablation; No visible MRI target	[44,51]
Clinical Stage	TNM stage: T1–T2 N0M0; Clinical stage ≤ T2b	Advanced local stage; Nodal or metastatic disease	[43,52]
Anatomical	Prostate volume > 40 mL acceptable	Tumors abutting urinary sphincter or urethra; Large prostatic calcifications	[44,52]
Diagnostic	Transperineal template saturation + MRI/TRUS fusion-targeted biopsies	Inadequate biopsy sampling or staging	[51]
Patient Factors	Age > 70 years (for whole-gland); unsuitable for or refusing radical therapy; prioritizing QoL preservation	Patient unable to tolerate transrectal procedure	[42,52]

Abbreviations: ISUP = International Society of Urological Pathology; PI-RADS = prostate imaging reporting and data system; NVB = neurovascular bundle; TRUS = transrectal ultrasound; QoL = quality of life.

**Table 8:** Published clinical trials of FUS ablation for primary breast cancer.

Author, Year	*N*	Mean Tumor Size	FUS Modality	Guidance	Key Finding
Wu et al., 2003 [55]	48 (23 FUS arm)	T1–T2	HIFU ablation	US	Complete coagulative necrosis; loss of PCNA, MMP-9, CD44v6 expression; upregulation of HSP70, CD3, CD4, CD8, NK cells vs. surgery alone
Wu et al., 2005 [56]	22	Mean 3.4 cm	HIFU ablation	US	Complete tumor necrosis in all; replacement by fibroglandular tissue by 6–12 months
Gianfelice et al., 2003 [59]	12	1.1–8.8 cm	MRgFUS ablation	MRI	Safe treatment; variable ablation coverage; all proceeded to surgical resection
Furusawa et al., 2006 [57]	30	Not reported	MRgFUS ablation	MRI	90% of patients had > 95% tumor ablated; residual tumor correlated with incomplete coverage
Furusawa et al., 2007 [58]	21	Not reported	MRgFUS ablation	MRI	Complete necrosis in 95% (20/21); 1 local recurrence detected reliably by contrast-enhanced MRI
Merckel et al., 2016 [60]	10	Not reported	MRgFUS ablation (dedicated breast platform)	MRI	Histopathologically proven tumor necrosis in 6/10; good correlation between applied energy and necrosis size; no complications

Abbreviations: US = ultrasound; PCNA = proliferating cell nuclear antigen; MMP-9 = matrix metalloproteinase-9; CD44v6 = CD44 variant 6; HSP70 = heat shock protein 70; NK = natural killer (cells) Adapted from Pediconi et al. [61]. Note: These studies are heterogeneous in design, sample size, endpoints, follow-up, adverse-event reporting, and treatment protocols. This table is intended as a compendium of reported and ongoing clinical experience rather than a head-to-head comparison. Please refer to each cited study for detailed methods and outcome definitions.

**Table 9:** Open and recently completed clinical trials of FUS in breast cancer.

Trial/NCT	Phase	Indication	FUS Modality	Adjunctive Therapy	Status
Breast 48 NCT03237572	Pilot/Phase I	Metastatic breast cancer (single lesion)	Subtotal HIFU ablation	Pembrolizumab (PD-1 blockade)	Terminated per Clinical trials.gov; early analyses ongoing [63]
Breast 54 NCT04796220	Phase I	Stage I–III breast cancer	Thermally ablative FUS	Low-dose gemcitabine	Actively recruiting [64]
BRIFU NCT03342625	Feasibility	Primary invasive ductal carcinoma ≤ 15 mm; treat-and-resect design	MRgFUS ablation	None (ablation monotherapy)	Actively recruiting (Institut Bergonié, France) [66]
NCT04431674 (Moore-Palhares)	Phase I	Stage I–IV BC; in situ tumors; radiotherapy indicated	MRgFUS-stimulated microbubbles (MRgFUS-MB)	External beam radiotherapy	Completed; published 2024 [65]

**Abbreviations:** MB = MR-guided focused ultrasound-stimulated microbubbles; PD-1 = programmed death-1. Note: These studies are heterogeneous in design, sample size, endpoints, follow-up, adverse-event reporting, and treatment protocols. This table is intended as a compendium of reported and ongoing clinical experience rather than a head-to-head comparison. Please refer to each cited study for detailed methods and outcome definitions.

## Data Availability

At the beginning of each section that provides the review for the use of FUS for a specific cancer type, the authors of each section declared the methods that were used to perform a literature search specific to the topic being discussed and included the benefits and limitations of their methods used and references chosen to cite.
